# Roadmap to safety: a single center study of evidence-informed approach to placenta accreta spectrum

**DOI:** 10.3389/fsurg.2024.1347549

**Published:** 2024-03-06

**Authors:** Rachel A. Levy, Prisca C. Diala, Harriet T. Rothschild, Jasmine Correa, Evan Lehrman, John C. Markley, Liina Poder, Joseph Rabban, Lee-may Chen, Jo Gras, Nasim C. Sobhani, Arianna G. Cassidy, Jocelyn S. Chapman

**Affiliations:** ^1^ Department of Obstetrics, Gynecology & Reproductive Sciences, University of California, San Francisco, CA, United States; ^2^ School of Medicine, University of California, San Francisco, CA, United States; ^3^ Department of Interventional Radiology, University of California, San Francisco, CA, United States; ^4^ Department of Anesthesia and Perioperative Care, University of California, San Francisco, CA, United States; ^5^ Department of Diagnostic Radiology, University of California, San Francisco, CA, United States; ^6^ Department of Pathology, University of California, San Francisco, CA, United States; ^7^ Division of Gynecologic Oncology, Department of Obstetrics, Gynecology & Reproductive Sciences, University of California, San Francisco, CA, United States; ^8^ Division of Maternal Fetal Medicine, Department of Obstetrics, Gynecology & Reproductive Sciences, University of California, San Francisco, CA, United States

**Keywords:** accreta spectrum, cesarean hysterectomy, multidisciplinary approach, uterine artery embolization, intraoperative complication

## Abstract

**Objective:**

To assess the impact of an evidence-informed protocol for management of placenta accreta spectrum (PAS).

**Methods:**

This was a retrospective cohort study of patients who underwent cesarean hysterectomy (c-hyst) for suspected PAS from 2012 to 2022 at a single tertiary care center. Perioperative outcomes were compared pre- and post-implementation of a standardized Multidisciplinary Approach to the Placenta Service (MAPS) protocol, which incorporates evidence-informed perioperative interventions including preoperative imaging and group case review. Intraoperatively, the MAPS protocol includes placement of ureteral stents, possible placental mapping with ultrasound, and uterine artery embolization by interventional radiology. Patients suspected to have PAS on prenatal imaging who underwent c-hyst were included in the analysis. Primary outcomes were intraoperative complications and postoperative complications. Secondary outcomes were blood loss, need for ICU, and length of stay. Proportions were compared using Fisher's exact test, and continuous variables were compared used *t*-tests and Mood's Median test.

**Results:**

There were no differences in baseline demographics between the pre- (*n* = 38) and post-MAPS (*n* = 34) groups. The pre-MAPS group had more placenta previa (95% pre- vs. 74% post-MAPS, *p* = 0.013) and prior cesarean sections (2 prior pre- vs. 1 prior post-MAPS, *p* = 0.012). The post-MAPS group had more severe pathology (PAS Grade 3 8% pre- vs. 47% post-MAPS, *p* = 0.001). There were fewer intraoperative complications (39% pre- vs.3% post-MAPS, *p* < 0.001), postoperative complications (32% pre- vs.12% post-MAPS, *p* = 0.043), hemorrhages >1l (95% pre- vs.65% post-MAPS, *p* = 0.001), ICU admissions (59% pre- vs.35% post-MAPS, *p* = 0.04) and shorter hospital stays (10 days pre- vs.7 days post-MAPS, *p* = 0.02) in the post-MAPS compared to pre-MAPS patients. Neonatal length of stay was 8 days longer in the post-MAPS group (9 days pre- vs. 17 days post-MAPS, *p* = 0.03). Subgroup analyses demonstrated that ureteral stent placement and uterine artery embolization (UAE) may be important steps to reduce complications and ICU admissions. When comparing just those who underwent UAE, patients in the post-MAPS group experienced fewer hemorrhages greater five liters (EBL >5l 43% pre- vs.4% post-MAPS, *p* = 0.007).

**Conclusion:**

An evidence-informed approach to management of PAS was associated with decreased complication rate, EBL >1l, ICU admission and length of hospitalization, particularly for patients with severe pathology.

## 1 Introduction

Abnormal placentation occurs when the placenta is partially or completely unable to detach from the uterus after delivery of a neonate. A standardized grading system for placenta accreta spectrum (PAS) has been endorsed by The International Federation of Gynecology and Obstetrics (FIGO), The Royal College of Obstetricians and Gynaecologists (RCOG), The American College of Obstetricians and Gynecologists (ACOG) and The Society for Maternal-Fetal Medicine (SMFM) (1). For the purposes of this manuscript, Grades 1, 2 and 3 to refer to previously accepted categories of pathologic findings of placenta accreta, placenta increta, and placenta percreta respectively. PAS is associated with significant maternal and neonatal risks including a 100-fold increase in maternal mortality compared to pregnancies unaffected by PAS (2), and is a leading driver of severe maternal morbidity and increased rates of premature delivery (3).

In the United States, there is a public health effort to decrease maternal mortality (4–7). Important to this effort is identification of strategies to effectively manage PAS, as rates of this disease have increased 5-fold over the last 40 years, along with increased rates of cesarean delivery (8, 9).

Management of PAS is not yet standardized, although prior research has identified important principles and interventions associated with improved outcomes.

Enhanced recovery after surgery (ERAS) protocols operate on the principle that no single intervention is sufficient to improve surgical recovery outcomes. While the ERAS® Society has developed cesarean section bundles for antepartum, intraoperative, and postpartum care, there are still no specific guidelines for cesarean hysterectomy (c-hyst) and PAS. Bundling multiple evidence-informed approaches, however, can have a dramatic impact (10, 11). Using this paradigm, our institutional Multidisciplinary Approach to Placenta Service (MAPS) developed a protocol incorporating evidence-informed interventions to standardize pre-, intra- and postoperative care for patients undergoing c-hyst for prenatally suspected PAS. This study compares maternal and perinatal outcomes before and after implementation of the MAPS protocol. We hypothesize that compared to the pre-MAPS group, the post-MAPS group has improved outcomes.

## 2 Methods

This is a retrospective cohort study of patients who underwent c-hyst for PAS before and after implementation of the MAPS protocol on January 1, 2018. The study was approved by the University of California, San Francisco institutional review board. Patients with ICD9 and 10 codes (043.21, 043.22, 043.32) for placenta accreta, placenta increta and placenta percreta between January 1, 2012, and December 31, 2017 were allocated to the pre-MAPS group, and patients who underwent MAPS consult and underwent c-hyst between January 1, 2018 and December 31, 2022 were allocated to the post-MAPS group. Patients were excluded if there was no suspicion for PAS on prenatal imaging or if c-hyst was performed for an indication other than PAS.

A nine-month period of overlap during which the protocol was implemented was considered a “washout period” to account for the elements of the protocol that occur from the time of referral to our center and delivery, i.e., those who underwent c-hyst immediately after implementation of the MAPS protocol in January 2018 would not have experienced all aspects of the protocol. Six patients underwent c-hyst during the “washout” period between January 1, 2018 and August 31, 2018 and were excluded from analysis.

**Figure 1:**
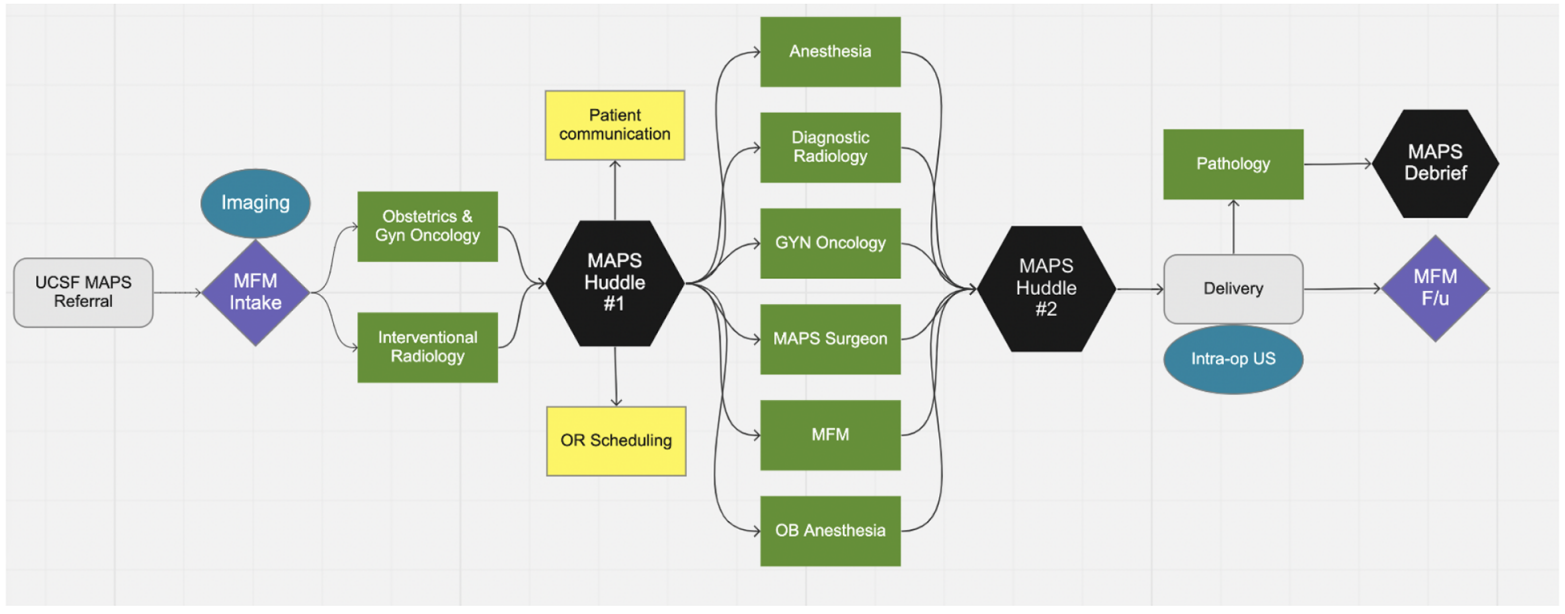
Multidisciplinary approach to the placenta service protocol flow.

Prior to MAPS implementation, PAS was managed without a standardized approach. After January 1st, 2018, all pregnant patients with PAS suspected on prenatal imaging were referred to the MAPS team and received treatment according to the protocol, which is summarized in Figure 1 and described in detail below.

The preoperative elements of the MAPS protocol begins after referral to our center. After initial consultation with Maternal Fetal Medicine (MFM), the patient undergoes ultrasound (US) and magnetic resonance imaging (MRI) of the placenta. Next, there is a multidisciplinary case review to finalize the delivery plan. Five general obstetricians have been trained to manage a subset of PAS without routine gynecologic oncology (gynonc) participation. Services such as gynecologic oncology, vascular surgery, urologic surgery, transfusion medicine, and interventional radiology are utilized as needed determined by the multidisciplinary case review. In our center, a “hybrid” operating suite, i.e., an operating suite with both conventional surgical equipment and interventional radiology equipment, is available, so that both procedures can be performed without the need to move the patient to a different room. There are currently two hybrid ORs at our institution. In the MAPS protocol, patients with PAS undergo c-hyst in hybrid OR unless prevented by patient acuity prevents this. No element of the MAPS protocol is required; the delivery plan is individualized after team discussion.

The intraoperative features of the MAPS protocol include routine placement of ureteral stents, placental mapping with ultrasound to determine location of the hysterotomy (i.e., hysterotomy placement in an area of the uterus where there is no underlying placenta), use of cell saver, and UAE by interventional radiology (IR) after delivery but before hysterectomy. Postoperative features of the protocol include use of ERAS pathway and prophylactic anticoagulation while inpatient given the combined risk for venous thromboembolism postpartum and after laparotomy (11, 12).

Final pathologic diagnosis is according to the international pathology grading system which defines Grade 1 as noninvasive, Grade 2 as superficial invasion and Grade 3 as deep invasion (1).

The following clinical data were extracted from the medical: demographics (height, weight, BMI, age, language, race, ethnicity, common medical comorbidities, and insurance status), prenatal ultrasound findings, delivery outcomes, perioperative details (surgical team, location of surgery, use of blood products, and delivery status), neonatal outcomes, and pathology diagnosis. Prior uterine procedures were defined as dilation and curettage (D&C), dilation and evacuation (D&E), myomectomy (abdominal, laparoscopic, hysteroscopic), other hysteroscopy, and endometrial ablation. Cases booked as standby (without delay, but not yet scheduled), within 48 h, within 24 h, within 6 h, urgent (within hours of decision to operate) and emergent (without delay) were all considered urgent. All other cases were considered scheduled.

The primary outcome was a composite of intraoperative complications, defined as urinary tract injury and vascular injury.

Secondary outcomes included a composite of postoperative complications, defined as disseminated intravascular coagulation (DIC) (defined as <200 mg/dl serum fibrinogen) (13), renal dysfunction (defined as serum creatinine increased two times the patient's baseline) (14), cardiac dysfunction (defined as ejection fraction less than 40%) (15), postpartum transfusion, pulmonary dysfunction (defined as pulmonary embolism, pulmonary infection, and need for postoperative intubation), re-operation within 1 week, re-admission within 1 month, infection (defined as class 2 or greater) (16) or maternal death within 6 weeks of delivery.

Other secondary maternal outcomes included estimated blood loss (EBL), need for blood transfusion, and maternal ICU admission.

Neonatal outcomes included a composite of neonatal complications (defined as respiratory distress syndrome, intraventricular hemorrhage, necrotizing enterocolitis and intubation); gestational age at delivery; birthweight; Apgar scores; neonatal ICU admission; and neonatal length of stay.

Univariate analysis was used to compare baseline characteristics. Two-tailed t-test was used to compare normally distributed continuous variables and two tailed Mood's Median test was used to compare continuous variables that were not normally distributed. Fisher's exact test was used to compare categorical variables. *p* < 0.05 was considered statistically significant for all comparisons. Statistical analyses were performed using Stata BE 17.0.

To assess the impact of ureteral stent placement, IR embolization, location of delivery, urgency of delivery, and severity of pathology, subgroup analyses were performed.

Because no patients in the pre-MAPS group delivered in the hybrid OR, the potential impact of the delivery in the hybrid OR was compared within the post-MAPS group to patients who delivered in a standard OR.

## 3 Results

78 patients met inclusion criteria: 38 (53%) in the pre-MAPS group and 34 (47%) in the post-MAPS group. Of 41 patients pre-MAPS and 44 patients post-MAPS, 3 and 4 patients (respectively) were referred but did not have suspected PAS on imaging so were excluded from further analysis. Six patients were excluded from the post-MAPS group to account for washout.

There were no significant changes in surgeons, surgical technique, anesthetic technique, or technology during the institution of the MAPS protocol.

There were no differences in basic demographics between the two groups (Table 1). The pre-MAPS group had higher gravidity (gravida 5 (IQR 4–6) pre- vs. 4 (IQR 2–4) post-MAPS, *p* = 0.002), parity (total para 2 (IQR 2–3) pre- vs.2 (IQR 1–2) post-MAPS, *p* = 0.004), and number of prior cesarean deliveries (2 (IQR 1–3) pre- vs.1 (IQR 1–2) post-MAPS, *p* = 0.012) (Table 1). A larger portion of the post-MAPS group, had at least one prior uterine (non-cesarean) procedure (21% pre- vs.44% post-MAPS, *p* = 0.036). The pre-MAPS group had a larger portion of placenta previa (95% pre- vs.74% post-MAPS, *p* = 0.013).

**Table 1:** Demographics of patients with PAS before and after implementation of MAPS.

	Pre-MAPS (*n* = 38)	Post-MAPS (*n* = 34)	*p* value
Maternal age in years	34 (32–37)	35 (31–40)	0.35
Race and ethnicity			0.66
Asian	2 (5%)	5 (15%)	
Black (not Latinx)	2 (5%)	1 (3)	
Latinx	17 (45%)	17 (50%)	
Other/Unknown	7 (18%)	5 (15%)	
White (not Latinx)	10 (26%)	6 (18%)	
Gravity	5 (4–6)	4 (2–4)	0.002^a^
Parity	2 (2–3)	2 (1–2)	0.004^a^
BMI (Kg/m^2^)	33 (25–38)	30 (26–34)	0.24
Nulliparous	0 (0%)	2 (6%)	0.13
Number of prior cesarean deliveries	2 (1–3)	1 (1–2)	0.012^a^
At least one prior uterine procedure	8 (21%)	15 (44%)	0.036^a^
Placenta previa	36 (95%)	25 (74%)	0.013^a^
Current multiple gestation	0 (0%)	0 (0%)	–
Diabetes	14 (37%)	9 (26%)	0.35
Hypertensive disease	4 (11%)	4 (12%)	0.87
Current IVF pregnancy	2 (5%)	5 (16%)	0.15
Final pathology			0.001^a^
Normal placenta	1 (3%)	1 (3%)	
PAS Grade 1	11 (29%)	9 (26%)	
PAS Grade 2	23 (61%)	7 (21%)	
PAS Grade 3	3 (8%)	16 (47%)	

Normally distributed data are presented as mean ± standard deviation, data that are not normally distributed are presented as median (interquartile range), and categorical data are presented as number (percentage).

BMI, body mass index; IVF, *in vitro* fertilization; MAPS, multidisciplinary approach to the placenta service; PAS, placenta accreta spectrum.

^a^ Statistically significant difference at *p* < 0.05.

Post-MAPS patients were more likely to have PAS Grade 3 on pathology examination of the surgical specimen (8 pre- vs. 47% post-MAPS, *p* = 0.001).

Post-MAPS patients were more likely to have intraoperative ultrasound (28% pre- vs.81% post-MAPS, *p* < 0.001) and uterine artery embolization (18% pre- vs.71% post-MAPS, *p* < 0.00) (Table 2). The distribution of scheduled and urgent deliveries was similar between groups.

**Table 2:** Perioperative management of patients with PAS before and after implementation of MAPS.

	Pre-MAPS (*n* = 38)	Post-MAPS (*n* = 34)	*p* value
Teams present intra-op			
Interventional radiology	7 (18%)	24 (71%)	<0.001^a^
Gynecologic oncology	33 (87%)	26 (76%)	0.25
Radiology	10 (28%)	26 (81%)	<0.001^a^
Urology	2 (6%)	3 (9%)	0.64
Vascular surgery	2 (6%)	0 (0%)	0.16
Delivery status			0.41
Scheduled	31 (82%)	25 (74%)	
Urgent	7 (18%)	9 (26%)	

Data that are not normally distributed are presented as median (interquartile range) and analyzed with Mood's Median test (two-tailed) and categorical data are presented as number (percentage) and analyzed with Fisher's exact test (two-tailed).

MAPS, multidisciplinary approach to the placenta service; PAS, placenta accreta spectrum; IR, interventional radiology; UAE, uterine artery embolization.

^a^ Statistically significant difference at *p* < 0.05.

### 3.1 Primary outcomes

After implementation of the MAPS protocol, the rate of intraoperative complications decreased from 39% to 3% (*p* < 0.001, Table 3). The most common intraoperative complication in both groups was cystotomy (12 of 15 pre- vs. 1 of 1 post-MAPS). The rate of postoperative complications also decreased from 32% to 12% (*p* = 0.043, Table 3).

**Table 3:** Primary maternal outcomes including rate of intraoperative and postoperative complications.

	Pre-MAPS (*n* = 38)	Post-MAPS (*n* = 34)	*p* value
Intraoperative complications	15 (39%)	1 (3%)	<0.001^a^
Postoperative complications	12 (32%)	4 (12%)	0.043^a^

Categorical data are presented as number (percentage) and analyzed with Fisher's exact test (two-tailed).

MAPS, multidisciplinary approach to the placenta service.

^a^ Statistically significant difference at *p* < 0.05.

### 3.2 Secondary outcomes

There were no differences in estimated blood loss (EBL), transfusion rates of all blood products or use of cell salvage. While the majority of surgeries had an EBL greater than 1liter pre-MAPS (95%), only 65% had EBL > 1l in the post-MAPS group (*p* = 0.001, Table 4). There were no differences between rates of 2l, 3l, 4l, or 5l blood loss between groups. The rate of ICU admission decreased from 59% pre- to 35% post-MAPS (*p* = 0.042, Table 4). ICU length of stay was similar between groups, but total hospital stay was shorter in the post-MAPS group (10 days (IQR 6–18) pre- vs.7 days (IQR 5–10) post-MAPS, *p* = 0.015, Table 4).

**Table 4:** Secondary maternal outcomes including blood loss, transfusion of blood products, level of care and length of stay.

	Pre-MAPS (*n* = 38)	Post-MAPS (*n* = 34)	*p* value
Estimated blood loss (EBL)	2,000 (1200–4000)	1,425 (700–3275)	0.13
EBL > 1l	36 (95%)	22 (65%)	0.001^a^
EBL > 2l	20 (53%)	16 (47%)	0.64
EBL > 4l	10 (26%)	7 (21%)	0.57
Received PRBC intra-op	24 (63%)	23 (68%)	0.69
Transfused >2u PRBC intra-op	27 (71%)	26 (76%)	0.60
Transfused >4u PRBC intra-op	25 (66%)	17 (50%)	0.17
Transfused >6u PRBC intra-op	21 (55%)	17 (50%)	0.66
Total transfused			
PRBC (units)	3 (1–9)	4 (1–8)	0.65
FFP (units)	3 (0–8)	2 (0–7)	0.44
Platelets (units)	0 (0–2.5)	0 (0–1)	0.36
Cryoprecipitate (units)	0 (0–0)	0 (0–0)	0.62
Cell salvage (ml)	500 (200–723)	363 (200–500)	0.23
Maternal ICU admission	22 (59%)	12 (35%)	0.04^a^
Maternal length of stay in days			
Total	10 (6–18)	7 (5–10)	0.02^a^
Postpartum	4 (4–5)	4 (4–5)	0.86
ICU	1 (1–2)	1 (1–2)	0.93

Data that are not normally distributed are presented as median (interquartile range) and analyzed with Mood's Median test (two-tailed) and categorical data are presented as number (percentage) and analyzed with Fisher's exact test (two-tailed).

MAPS, multidisciplinary approach to the placenta service; PAS, placenta accreta spectrum; L, Liters; u, unit; PRBC, packed red blood cells; FFP, fresh frozen plasma; ICU, intensive care unit.

^a^ Statistically significant difference at *p* < 0.05.

### 3.3 Subgroup analysis

We examined the effect of the MAPS protocol on those who underwent scheduled and urgent c-hyst. The MAPS protocol was associated with reduction in intraoperative complications in both groups: 31% reduction among scheduled (*p* = 0.004) and a 57% reduction among urgent (*p* = 0.009) (Table 4). Among scheduled cases there was a reduction in intraoperative hemorrhage (EBL >1l) from 97% pre- to 60% (*p* < 0.001, Table 4).

We examined the effect of the MAPS protocol on subgroups of those with PAS Grade 1 and those with PAS Grades 2–3. Among those with less severe pathology, implementation of the MAPS protocol was associated with decreased blood loss (EBL >1l 100% pre- vs. 67% post-MAPS, *p* = 0.038) and decreased need for greater than 4 units of PRBC (91% pre- vs. 33% post-MAPS, *p* = 0.007, Table 5). Those with more severe pathology experienced elimination of intraoperative complications (50% pre- vs. 0% post-MAPS, *p* < 0.001), decreased blood loss (EBL >1l 92% pre- vs. 70% post-MAPS, *p* = 0.04), and decreased need for maternal ICU admission (72% pre- vs. 35% post-MAPS, *p* = 0.01, Table 5).

**Table 5:** Subgroup analysis of select primary and secondary outcomes including complications, estimated blood loss (EBL) > 1l, transfusion > 4 units of packed red blood cells (pRBC), intensive care unit (ICU) admission before and after implementation of the MAPS protocol.

5a. Scheduled procedures only *N* (% of subgroup, % of total Pre or Post)	Pre-MAPS (*n* = 31)	Post-MAPS (*n* = 25)	*p* value
Intraoperative complications	11 (35%, 29% total)	1 (4%, 3% total)	0.004^a^
Postoperative complications	10 (32%, 26% total)	3 (12%, 9% total	0.07
EBL > 1L	30 (97%, 79% total)	15 (60%, 44% total)	<0.001^a^
Transfused > 4u PRBC	19 (61%, 50% total)	13 (52%, 38% total)	0.48
Maternal ICU admission	18 (58%, 47% total)	9 (36%, 26% total)	0.10
5b. Urgent procedures only *N* (% of subgroup, % of total Pre or Post)	Pre-MAPS (*n* = 7)	Post-MAPS (*n* = 9)	
Intraoperative complications	4 (57%, 11% total)	0 (0%, 0% total)	0.009^a^
Postoperative complications	2 (29%, 5% total)	1 (11%, 3% total)	0.37
EBL > 1L	6 (86%, 16% total)	7 (78%, 21% total)	0.69
Transfused > 4u PRBC	6 (86%, 16% total)	4 (44%, 12% total)	0.09
Maternal ICU admission	4 (57%, 11% total)	3 (33%, 9% total)	0.20
5c. PAS Grade 1 *N* (% of subgroup, % of total Pre or Post)	Pre-MAPS (*n* = 11)	Post-MAPS (*n* = 9)	
Intraoperative complications	2 (18%, 5% total)	1 (11%, 3% total)	0.66
Postoperative complications	3 (27%, 8% total)	0 (0%, 0% total)	0.89
EBL > 1L	11 (100%, 29% total)	6 (67%, 18% total)	0.038^a^
Transfused > 4u PRBC	10 (91%, 26% total)	3 (33%, 9% total)	0.007^a^
Maternal ICU admission	3 (27%, 8% total)	4 (44%, 12% total)	0.42
5d. PAS Grade 2 or 3 *N* (% of subgroup, % of total Pre or Post)	Pre-MAPS (*n* = 26)	Post-MAPS (*n* = 23)	
Intraoperative complications	13 (50%, 34% total)	0 (0%, 0% total)	<0.001^a^
Postoperative complications	9 (35%, 24% total)	4 (17%, 12% total)	0.17
EBL > 1L	24 (92%, 63% total)	16 (70%, 47% total)	0.04^a^
Transfused > 4u PRBC	15 (58%, 39% total)	12 (52%, 35% total)	0.70
Maternal ICU admission	18 (72%, 47% total)	8 (35%, 24% total)	0.01^a^
5e. Ureteral stents placed *N* (% of subgroup, % of total Pre or Post)	Pre-MAPS (*n* = 8)	Post-MAPS (*n* = 32)	
Intraoperative complications	4 (50%, 11% total)	0 (0%, 0% total)	<0.001^a^
Postoperative complications	2 (25%, 5% total)	4 (12%, 12% total)	0.38
EBL > 1L	8 (100%, 21% total)	20 (62%, 35% total)	0.04^a^
Transfused > 4u PRBC	5 (62%, 13% total)	17 (53%, 50% total)	0.63
Maternal ICU admission	6 (75%, 16% total)	11 (34%, 32% total)	0.04^a^
5f. Cystoscopy performed *N* (% of subgroup, % of total Pre or Post)	Pre-MAPS (*n* = 14)	Post-MAPS (*n* = 33)	
Intraoperative complications	7 (50%, 18% total)	1 (3%, 3% total)	<0.001^a^
Postoperative complications	3 (21%, 8% total)	4 (12%, 12% total)	0.41
EBL > 1L	13 (93%, 34% total)	22 (67%, 65% total)	0.06
Transfused > 4u PRBC	6 (43%, 16% total)	17 (52%, 50% total)	0.59
Maternal ICU admission	10 (71%, 26% total)	12 (36%, 35% total)	0.03^a^
5g. Underwent UAE *N* (% of subgroup, % of total Pre or Post)	Pre-MAPS (*n* = 7)	Post-MAPS (*n* = 24)	
Intraoperative complications	3 (43%, 8% total)	0 (0%, 0% total)	<0.001^a^
Postoperative complications	5 (71%, 13% total)	3 (12%, 9% total)	0.002^a^
EBL > 1L	5 (71%, 13% total)	12 (50%, 35% total)	0.32
EBL > 5L	3 (43%, 8% total)	1 (4%, 3% total)	0.007^a^
Transfused > 4u PRBC	5 (71%, 13% total)	12 (50%, 35% total)	0.32
Maternal ICU admission	5 (71%, 13% total)	5 (21%, 15% total)	0.01^a^

Categorical data are presented as number (percentage) and analyzed with Fisher's exact test (two-tailed).

MAPS, multidisciplinary approach to the placenta service; L, Liters; u, unit; PRBC, packed red blood cells; FFP, fresh frozen plasma; ICU, intensive care unit; UAE, uterine artery embolization.

^a^ Statistically significant difference at *p* < 0.05.

Compared to pre-MAPS, post-MAPS patients were more likely to undergo cystoscopy (37% vs. 97%), ureteral stent placement (21% vs. 94%), and UAE (18% vs. 80%). We compared those who underwent these intra-operative procedures pre- and post-MAPS. Those who underwent any of these intra-operative procedures experienced fewer intra-operative complications: cystoscopy 50% pre- vs.3% post-MAPS (*p* < 0.001); stent placement 50% pre- vs.0% post-MAPS (*p* < 0.001); UAE 43% pre- vs.0% post-MAPS (*p* = 0.01). The same group had a decreased rate of maternal ICU admission: cystoscopy 71% pre- vs.36% post-MAPS (*p* < 0.03); stent placement 75% pre- vs.11% post-MAPS (*p* = 0.04); UAE 71% pre- vs.21% post-MAPS (*p* < 0.01) (Table 5). Those who underwent UAE after implementation experienced fewer postoperative complications (71% vs.12%, *p* = 0.002) compared to those who underwent UAE prior to implementation (Table 5). Although those who underwent UAE did not experience decrease in hemorrhage >1l, they were less likely to have more severe hemorrhage (EBL >5l 43% pre- vs.4% post-MAPS, *p* = 0.007, Table 5).

### 3.4 Neonatal outcomes

There were 33 neonates in the post-MAPS group (one in the post-MAPS group had an intrauterine fetal demise prior to delivery). There were no differences in neonatal complications between the two groups. There was a trend toward earlier gestational age at time of delivery post-MAPS, though this was not statistically significant. There was a decrease in 5-min Apgar score 8 (IQR 6–9) pre- vs.6 (IQR 4–8) post-MAPS (*p* = 0.03), though no difference in 1 and 10 min Apgar scores. The length of stay for the neonate increased 9 days (IQR 5–18) pre- to 17 days (IQR 7–25) post-MAPS (*p* = 0.03, Table 6).

**Table 6:** Neonatal outcomes before and after implementation of MAPS protocol.

	Pre-MAPS (*n* = 38)	Post-MAPS (*n* = 33)^b^	*p* value
Birthweight in grams	2,435 (2200–2810)	2,230 (1970–2505)	0.07
Gestational age at time of delivery in weeks	35 (34–35)	34 (33–35)	0.21
Apgars			
1 min	6 (3–7)	4 (2–7)	0.18
5 min	8 (6–9)	6 (4–8)	0.03^a^
10 min	7 (7–8)	7 (5.5–8)	0.68
Neonatal ICU admission	33 (87%)	32 (97%)	0.13
Neonatal length of stay	9 (5–18)	17 (7–25)	0.03^a^
Neonatal complications			
Respiratory distress syndrome	16 (42%)	21 (64%)	0.072
Intraventricular hemorrhage	1 (3%)	0 (0%)	0.63
Necrotizing enterocolitis	0 (0%)	0 (0%)	–
Intubation	4 (11%)	6 (19%)	0.33

Data that are not normally distributed are presented as median (interquartile range) and analyzed with Mood's Median test (two-tailed) and categorical data are presented as number (percentage) and analyzed with Fisher's exact test (two-tailed).

MAPS, multidisciplinary approach to the placenta service; ICU, intensive care unit.

^a^ Statistically significant difference at *p* < 0.05.

^b^ After implementation of the MAPS protocol, one patient experienced intrauterine fetal demise. Therefore, there are 33 neonates in the post-MAPS group.

## 4 Discussion

At a single tertiary care center, implementation of an evidence-based multidisciplinary team protocol approach led to a reduction in perioperative complications, postpartum hemorrhage, ICU admission, and length of maternal hospitalization. Our collective optimism about evidence-based protocols for management of PAS is inextricable from the real morbidity and mortality associated with PAS.

A strength of this study was inclusion of a standardized approach in a single center with uniform reporting of prenatal imaging findings and pathology results. A challenge of multi-center studies of PAS is heterogeneity of reporting of imaging and pathology findings, which introduces challenges to standardization.

The retrospective nature of this study and small sample size are limitations to this study.

While our sample size is sufficient for study power, the two cohorts do not perfectly match. Both groups have equivalent total parity, though interquartile ranges were different between groups (P2 (IQR 2–3) pre- vs.P2 (IQR 1–2) post-MAPS, *p* = 0.004, Table 1). Therefore, those in the pre-MAPS group may have been at slightly increased risk for uterine atony and therefore hemorrhage. Patients in the pre-MAPS group had a higher number of prior cesarean deliveries (2 prior (IQR 1–3) pre- vs.1 prior (IQR 1–2) post-MAPS, *p* = 0.012, Table 1). This may have predisposed them to intraabdominal adhesions leading to intraoperative injury.

Placenta previa was more common in the pre-MAPS group (previa 95% pre- vs.74% post-MAPS, *p* = 0.013) and PAS Grade 3 was more common in the post-MAPS group (PAS Grade 3 8% pre- vs.47% post-MAPS, *p* = 0.001, Table 1). This may reflect increased awareness about other risk factors for PAS. Without logistic regression adjusting for these differences, the impact of the MAPS protocol is uncertain. We argue, however, that the impact of the differences in obstetric history (higher parity and number of prior cesarean sections for pre-MAPS patients) may be counteracted by the impact of the difference in gynecologic history, as there was a higher prevalence of prior uterine procedure in post-MAPS patients (44%) compared to that in pre-MAPS patients (21%) (*p* = 0.036, Table 1).

The study does not consider complications from IR (such as hematoma, aneurysm, pseudo-aneurysm) and anesthesia (such as airway complications), which is a limitation as the use of UAE increased post-MAPS (Table 5G).

PAS can require urgent clinical intervention. Despite antenatal evaluation, 20%–25% of patients in this study required urgent or emergent delivery. The similar intraoperative complication rates for urgent and scheduled cases post-MAPS highlight the adaptability of this protocol, allowing for streamlined team mobilization and surgical planning without jeopardizing patient outcomes.

As clinicians think more broadly about those at risk for PAS, it is possible that referrals increased for patients with a history of a uterine procedure that disrupts the endometrium (18). While we know that the ability of the placenta to adhere and invade the myometrium and surrounding pelvic organs involves a combination of uterine and placental factors, the specific defects in angiogenesis, proliferation and inflammation/invasion are not yet well understood (19). As such, it is possible that PAS Grade 2 in a uterus with multiple prior D&Cs may be clinically different than the PAS Grade 2 in a uterus with a prior cesarean section scar (20). If so, the pre- and post-MAPS groups may have fundamentally different pathologies that could account for the observed differences in outcomes.

Without logistic regression by gestational age at delivery, the reason for increased neonatal length of stay after the implementation of our protocol is unclear. Patients in the post-MAPS group may have delivered at an earlier gestational age because of close antepartum surveillance and delivery planning compared to patients without close antepartum surveillance. Further study is needed to evaluate this trend.

The complications evaluated in this study were those occurring in the acute postpartum period (21). Rates of delayed postpartum complications, particularly postpartum readmission, may be underestimated. Because we are a referral center, patients who live far away may present to a local hospital after discharge. While outside facilities often contact us for readmission of patients who underwent surgery at our institution, it is possible that not all complications are captured.

Conservative management strategies were not incorporated into our MAPS protocol for patients with a diagnosis of PAS. Uterine-sparing management of PAS is associated with complications such as sepsis and disseminated intravascular coagulation (DIC) and a recurrence risk for PAS in subsequent pregnancies exceeding 60% (22). The Placenta Accreta (PACCRETA) study reported lower rates of blood transfusion but higher rates of arterial embolus, readmission and infection in those managed conservatively (23). Uterine-sparing surgical techniques, however, including temporary occlusion of the internal iliac arteries have been presented with promising outcomes (24, 25). Patients referred to our program who, after imaging and team discussion, were eligible for a uterine sparing approach (i.e., preoperative imaging was not sufficiently suspicious for PAS) and underwent vaginal or cesarean delivery were not included in analysis. The impact of the MAPS protocol on this group of patients is not represented in this study.

Individuals who underwent a cesarean hysterectomy emergently without MAPS team evaluation and discussion were also not included. It is possible that the existence of the MAPS protocol and the systems in place to support a team-based approach may improve outcomes in this group even in the absence of a systematic preoperative MAPS evaluation.

This study builds on the pre-existing evidence regarding interventions for patients with PAS. Two cohort studies published more than ten years ago demonstrated decreased transfusion rates and reoperation rates in patients managed at high-volume centers and advocated for a multidisciplinary approach. Minimizing placental manipulation has been demonstrated to decrease blood loss (26).

Multiple interventional radiologic procedures have been employed and in small case series are associated with variable rates of decreased bleeding and post-procedure complications (27–30). In our institution, we have previously demonstrated gelfoam uterine artery embolization (UAE) following cesarean delivery but before hysterectomy in patients with PAS is associated with decreased EBL, transfusion requirements, and length of ICU stay compared with c-hyst alone (31). Other techniques to control intraoperative blood loss including temporary vascular occlusion of internal iliac arteries with clamps are not routinely part of our surgical protocol (24, 25, 32). Resuscitative endovascular balloon occlusion of the aorta (REBOA), which has also been studied for PAS surgery, is not available at our institution.

While ureteral stent placement was demonstrated to decrease ureteral injury in one study, it had no impact in another (33, 34). The use of intraoperative cell salvage can decrease the need for transfusion of allogenic blood products, which can be associated with transfusion reaction and infection (35). One retrospective cohort study and several case reports demonstrate the feasibility and safety of PAS management in a hybrid OR rather than in a traditional OR (36, 37). Several contemporary cohort studies evaluating single-institution, multidisciplinary management of PAS demonstrated decreased blood loss without increased intraoperative complications, improved neonatal Apgar scores and fewer emergent deliveries (38–40).

As with all bundled care protocols, our protocol includes multiple interventions, and no single intervention can be highlighted as the most important contributor to the observed decrease in morbidity. Bundled interventions can make for a costly and labor-intensive clinical program that warrants ongoing evaluation and even de-escalation when appropriate.

While gynecologic oncologists continue to navigate their role in managing PAS, institutions worldwide rely on gynecologic oncology at time of c-hyst for PAS (17). An important feature of our protocol was focused surgical training of a group of general obstetricians to manage PAS without routine gynecologic oncology participation. Implementation of our protocol may decrease the need for gynecologic oncology presence in the operating room at time of delivery. Perhaps with more streamlined detection and management of PAS as well as more frequent radiology and IR presence, the hospital system can more efficiently utilize surgical resources.

This study adds to the literature supporting an evidence-informed approach to caring for patients with PAS and suggests that such programs include evidence-informed pre-, intra-, and postoperative interventions to minimize the morbidity of this disease. With ongoing attention to patients with PAS and continued initiative to minimize the impact of this potentially devastating disease, we hope to curtail the ominous impact of PAS on maternal well-being.

## Data Availability

The raw data supporting the conclusions of this article will be made available by the authors, without undue reservation.

## References

[1] Jauniaux E Hussein AM Fox KA Collins SL. New evidence-based diagnostic and management strategies for placenta accreta spectrum disorders. Best Pract Res Clin Obstet Gynaecol. (2019) 61:75–88. 10.1016/j.bpobgyn.2019.04.006 31126811 PMC6929563

[2] Cahill AG Beigi R Heine RP Silver RM Wax JR. Placenta accreta spectrum. Am J Obstet Gynecol. (2018) 219(6):B2–B16. 10.1016/j.ajog.2018.09.042 30471891

[3] Vinograd A Wainstock T Mazor M Beer-Weisel R Klaitman V Dukler D Placenta accreta is an independent risk factor for late pre-term birth and perinatal mortality. J Matern Fetal Neonatal Med. (2015) 28(12):1381–7. 10.3109/14767058.2014.955004 25142109

[4] Howell EA. Reducing disparities in severe maternal morbidity and mortality. Clin Obstet Gynecol. (2018) 61(2):387–99. 10.1097/GRF.0000000000000349 29346121 PMC5915910

[5] Hoyert D. Maternal Mortality Rates in the United States, 2020. National Center for Health Statistics (U.S.). NCHS Health E-Stats (2022). 10.15620/cdc:113967 35947824

[6] Douthard RA Martin IK Chapple-McGruder T Langer A Chang SUS. Maternal mortality within a global context: historical trends, current state, and future directions. J Womens Health. (2021) 30(2):168–77. 10.1089/jwh.2020.8863 PMC8020556 33211590

[7] Eller AG Bennett MA Sharshiner M Masheter C Soisson AP Dodson M Maternal morbidity in cases of placenta accreta managed by a multidisciplinary care team compared with standard obstetric care. Obstet Gynecol. (2011) 117(2):331–7. 10.1097/AOG.0b013e3182051db2 21309195

[8] Wright JD Silver RM Bonanno C Gaddipati S Lu YS Simpson LL Practice patterns and knowledge of obstetricians and gynecologists regarding placenta accreta. J Matern Fetal Neonatal Med. (2013) 26(16):1602–9. 10.3109/14767058.2013.793662 23565991

[9] Belfort MA. Placenta accreta. Am J Obstet Gynecol. (2010) 203(5):430–9. 10.1016/j.ajog.2010.09.013 21055510

[10] Afessa B Gajic O Keegan MT Seferian EG Hubmayr RD Peters SG. Impact of introducing multiple evidence-based clinical practice protocols in a medical intensive care unit: a retrospective cohort study. BMC Emerg Med. (2007) 7(1):10. 10.1186/1471-227X-7-10 17686165 PMC1965465

[11] Chapman JS Roddy E Ueda S Brooks R Lynn CL May CL. Enhanced recovery pathways for improving outcomes after minimally invasive gynecologic oncology surgery. Obstet Gynecol. (2016) 128(1):138–44. 10.1097/AOG.0000000000001466 27275797

[12] Bates SM Greer IA Middeldorp S Veenstra DL Prabulos AM Vandvik PO. VTE, thrombophilia, antithrombotic therapy, and pregnancy. Chest. (2012) 141(2):e691S–736S. 10.1378/chest.11-2300 22315276 PMC3278054

[13] Cunningham FG Nelson DB. Disseminated intravascular coagulation syndromes in obstetrics. Obstet Gynecol. (2015) 126(5):999–1011. 10.1097/AOG.0000000000001110 26444122

[14] Lopes JA Jorge S. The RIFLE and AKIN classifications for acute kidney injury: a critical and comprehensive review. Clin Kidney J. (2013) 6(1):8–14. 10.1093/ckj/sfs160 27818745 PMC5094385

[15] Ponikowski P Voors AA Anker SD Bueno H Cleland JGF Coats AJS 2016 ESC guidelines for the diagnosis and treatment of acute and chronic heart failure: the task force for the diagnosis and treatment of acute and chronic heart failure of the European society of cardiology (ESC)Developed with the special contribution of the heart failure association (HFA) of the ESC. Eur Heart J. (2016) 37(27):2129–200. 10.1093/eurheartj/ehw128 27206819

[16] Onyekwelu I Yakkanti R Protzer L Pinkston CM Tucker C Seligson D. Surgical wound classification and surgical site infections in the orthopaedic patient. JAAOS Glob Res Rev. (2017) 1(3):e022. 10.5435/JAAOSGlobal-D-17-00022 PMC6132296 30211353

[17] Matsuo K Vestal NL Rau AR Sangara RN Youssefzadeh AC Bainvoll L Gynecologic oncologists in surgery for placenta accreta spectrum: a survey for practice, experience, and interest. Int J Gynecol Cancer. (2022) 32(11):1433–42. 10.1136/ijgc-2022-003830 36167437

[18] Morlando M Collins S. Placenta accreta spectrum disorders: challenges, risks, and management strategies. Int J Womens Health. (2020) 12:1033–45. 10.2147/IJWH.S224191 33204176 PMC7667500

[19] Bartels HC Postle JD Downey P Brennan DJ. Placenta accreta spectrum: a review of pathology, molecular biology, and biomarkers. Dis Markers. (2018) 2018:1–11. 10.1155/2018/1507674 PMC6051104 30057649

[20] Hessami K Salmanian B Einerson BD Carusi DA Shamshirsaz AA Shainker SA Clinical correlates of placenta accreta spectrum disorder depending on the presence or absence of placenta previa: a systematic review and meta-analysis. Obstet Gynecol. (2022) 140(4):599–606. 10.1097/AOG.0000000000004923 36075058

[21] Romano M Cacciatore A Giordano R La Rosa B. Postpartum period: three distinct but continuous phases. J Prenat Med. (2010) 4(2):22–5. . 22439056 PMC3279173

[22] Sentilhes L Seco A Azria E Beucher G Bonnet MP Branger B Conservative management or cesarean hysterectomy for placenta accreta spectrum: the PACCRETA prospective study. Am J Obstet Gynecol. (2022) 226(6):839.e1–839.e24. 10.1016/j.ajog.2021.12.013 34914894

[23] Timmermans S van Hof AC Duvekot JJ. Conservative management of abnormally invasive placentation. Obstet Gynecol Surv. (2007) 62(8):529–39. 10.1097/01.ogx.0000271133.27011.05 17634154

[24] Daggez M Aslanca T Dursun P. Intraoperative temporary internal iliac arterial occlusion (Polat’s technique) for severe placenta accreta spectrum: a description of the technique and outcomes in 61 patients. Int J Gynecol Obstet. (2024) 164(1):99–107. 10.1002/ijgo.14968 37377184

[25] Yang Z Yang Y Yin Z Yao J. The role of internal iliac artery intraoperative vascular clamp temporary occlusion in abnormally invasive placenta. Int J Gynecol Obstet. (2023) 161(1):175–81. 10.1002/ijgo.14422 35986614

[26] Berhan Y Urgie T. A literature review of placenta accreta spectrum disorder: the place of expectant management in Ethiopian setup. Ethiop J Health Sci. (2020) 30(2):277–92. 10.4314/ejhs.v30i2.16 32165818 PMC7060376

[27] Soyer P Barat M Loffroy R Barral M Dautry R Vidal V The role of interventional radiology in the management of abnormally invasive placenta: a systematic review of current evidences. Quant Imaging Med Surg. (2020) 10(6):1370–91. 10.21037/qims-20-548 32550143 PMC7276355

[28] Wei X Zhang J Chu Q Du Y Xing N Xu X Prophylactic abdominal aorta balloon occlusion during caesarean section: a retrospective case series. Int J Obstet Anesth. (2016) 27:3–8. 10.1016/j.ijoa.2015.12.001 26775894

[29] Minas V Gul N Shaw E Mwenenchanya S. Prophylactic balloon occlusion of the common iliac arteries for the management of suspected placenta accreta/percreta: conclusions from a short case series. Arch Gynecol Obstet. (2015) 291(2):461–5. 10.1007/s00404-014-3436-9 25178185

[30] Ordoñez CA Manzano-Nunez R Parra MW Rasmussen TE Nieto AJ Herrera-Escobar JP Prophylactic use of resuscitative endovascular balloon occlusion of the aorta in women with abnormal placentation: a systematic review, meta-analysis, and case series. J Trauma Acute Care Surg. (2018) 84(5):809–18. 10.1097/TA.0000000000001821 29401189

[31] Wang M Ballah D Wade A Taylor AG Rizzuto G Li B Uterine artery embolization following cesarean delivery but prior to hysterectomy in the management of patients with invasive placenta. J Vasc Interv Radiol. (2019) 30(5):687–91. 10.1016/j.jvir.2018.12.007 30922797 PMC10468213

[32] Dursun P. Use of bulldog vascular clamps to reduce intraoperative bleeding during cesarean hysterectomy for placenta percreta. Int J Gynecol Obstet. (2018) 140(3):379–80. 10.1002/ijgo.12302 28833082

[33] Scaglione MA Allshouse AA Canfield DR Mclaughlin HD Bruno AM Hammad IA Prophylactic ureteral stent placement and urinary injury during hysterectomy for placenta accreta Spectrum. Obstet Gynecol. (2022) 140(5):806–11. 10.1097/AOG.0000000000004957 36201777 PMC10069290

[34] Crocetto F Esposito R Saccone G Della Corte L Sarno L Morlando M Use of routine ureteral stents in cesarean hysterectomy for placenta accreta. J Matern Fetal Neonatal Med. (2021) 34(3):386–9. 10.1080/14767058.2019.1609935 30999793

[35] Carless PA Henry DA Moxey AJ O’Connell D Brown T Fergusson DA. Cell salvage for minimising perioperative allogeneic blood transfusion. In: The Cochrane Collaboration, editors. Cochrane Database of Systematic Reviews. John Wiley & Sons, Ltd (2010). p. CD001888.pub3. 10.1002/14651858.CD001888.pub3 17054147

[36] Meller CH Garcia-Monaco RD Izbizky G Lamm M Jaunarena J Peralta O Non-conservative management of placenta accreta spectrum in the hybrid operating room: a retrospective cohort study. Cardiovasc Intervent Radiol. (2019) 42(3):365–70. 10.1007/s00270-018-2113-y 30413916

[37] Lim ZW Lee WY Huang YC Wu WJ Chen M. Comparison of one-stage and two-stage intraoperative uterine artery embolization during cesarean delivery for placenta accreta: report of two clinical cases at a tertiary referral medical center. Healthcare. (2022) 10(5):774. 10.3390/healthcare10050774 35627911 PMC9141000

[38] Melber DJ Berman ZT Jacobs MB Picel AC Conturie CL Zhang-Rutledge K Placenta accreta spectrum treatment with intraoperative multivessel embolization: the PASTIME protocol. Am J Obstet Gynecol. (2021) 225(4):442.e1–442.e10. 10.1016/j.ajog.2021.07.001 34245679

[39] Sandlin AT Magann EF Whittington JR Schneider AM Ramseyer AM Hughes DS Management of pregnancies complicated by placenta accreta spectrum utilizing a multidisciplinary care team in a rural state. J Matern Fetal Neonatal Med. (2022) 35(25):5964–9. 10.1080/14767058.2021.1903425 33769169

[40] Shamshirsaz AA Fox KA Salmanian B Diaz-Arrastia CR Lee W Baker BW Maternal morbidity in patients with morbidly adherent placenta treated with and without a standardized multidisciplinary approach. Am J Obstet Gynecol. (2015) 212(2):218.e1–e9. 10.1016/j.ajog.2014.08.019 25173187

